# Advanced Research on Animal Venoms in China

**DOI:** 10.3390/toxins15040272

**Published:** 2023-04-06

**Authors:** Ren Lai, Qiumin Lu

**Affiliations:** Key Laboratory of Animal Models and Human Disease Mechanisms of the Chinese Academy of Sciences/Key Laboratory of Bioactive Peptides of Yunnan Province, Kunming Institute of Zoology, Kunming 650223, China

For millennia, scientists, researchers, and the general public have been intrigued by animal venoms due to their potent effects and paradoxical ability to both harm and heal. Animal venoms are complex cocktails of bioactive molecules produced and delivered by various animal species, including snakes, spiders, scorpions, toads, cone snails, centipedes, and even some mammals such as tree shrews and platypi. These molecules are incredibly diverse and specialized, with unique properties that allow venomous animals to capture prey, protect themselves, and compete for resources. Furthermore, many of these toxins have evolved to interact with specific molecular targets (i.e., ion channels) in the nervous, cardiovascular, and musculoskeletal systems of prey or predators. Due to their high potency and specificity to molecular targets, animal venoms are valuable tools for researching ion channel functions and related diseases, which can inform the development of new drugs and therapeutic strategies.

Recently, Chinese researchers have conducted significant work in animal venom research to comprehend the diversity and complexity of these molecules and their potential applications in medicine, biotechnology, and as research tools. Advancements in ‘omics’ technologies such as proteomics, genomics, and transcriptomics have empowered Chinese researchers to identify and analyze venom-coded genes and proteins, revealing a kaleidoscope of novel peptide toxins with diverse functions and activities.

In this Special Issue of *Toxins*, primary research papers have been assembled that provide the reader with a comprehensive and up-to-date perspective on some of the most recent and dynamic contributions of animal toxins research by Chinese researchers. The purpose of this issue is to provide the latest work by Chinese researchers on the discovery of animal toxins, the mechanism underlining their actions, the mining of drug leads from peptide toxins, and the diagnosis and treatment of bites or stings by venomous animals. Below is a brief synopsis of the 11 papers that make up this Special Issue.

Skin secretions from amphibians contain toxin-like proteins and peptides that play an important role in their physiological and pathological functions. Qingqing Ye and colleagues [1] have reported that βγ-CAT, a Chinese red-belly toad-derived pore-forming toxin-like protein complex, could induce various toxic effects via its membrane perforation process, including membrane binding, oligomerization, and endocytosis. This research reveals a hitherto unrecognized toxicological role of a vertebrate-derived pore-forming toxin-like protein in the nervous system, which causes hippocampus neuronal cells to undergo pyroptosis, which in turn results in cognitive retardation. Additionally, a study conducted by Chuanling Yin et al. [2] has focused on the secretions of tree frogs in order to gain insight into a unique defense mechanism in amphibians. The team has reported the presence of PAX in the secretions of tree frogs (*Hyla japonica*). There is a biological significance to PAX as it inhibits both BKCa and KCNK18 channels, which fire excitatory currents in sensory neurons, causing predators or competitors to feel tingly and buzzy. The presence of bifunctional PAX in frog skin secretion may indicate a unique defensive mechanism that is involved in amphibian adaptation. Moreover, a novel peptide, PM-7, from the frog Polypedates megacephalus has been identified by Siqi Fu and colleagues [3]. The authors demonstrate that the peptide has the capacity to promote wound healing in mice. PM-7 may be a potential candidate in the development of cutting-edge drugs for treating wounds.

The neurotoxic and myotoxic phospholipase A2 toxins found in the venom of Russell’s viper (*Daboia siamensis*) can potentially harm motor nerve terminals irreversibly. Antivenoms may only be partially effective against some of these venom components because of the time interval between envenoming and the delivery of the antivenom. Mimi Lay et al. [4] have assessed the effectiveness of Chinese *D. siamensis* antivenom alone and in conjunction with a PLA2 inhibitor, Varespladib, in restoring the in vitro neuromuscular blockade in the chick biventer cervicis nerve-muscle preparation. The authors demonstrate that the antivenom has a shorter window of effectiveness than Varespladib, and that some of the effects of Chinese *D. siamensis* venom may be inhibited by small-molecule inhibitors.

Fan Zhao and colleagues [5] have investigated the key factors responsible for the adverse effects caused by an analgesic-antitumor β-scorpion toxin (AGAP) from scorpion venom on human voltage-gated sodium channels 1.4 and 1.5. Their research explains the mechanism by which the tremendous modification of subtype selectivity might result from the mutation of a single amino acid. This work advances the development of safer and more effective therapies specifically targeting VGSC subtypes and preventing muscle and myocardium toxicity.

The peptides derived from scorpion venom have garnered significant interest as potential anticancer agents due to their unique properties, such as their high specificity and potency toward cancer cells. The anticancer mechanism of Smp24, a peptide derived from the venom of *Scorpio Maurus palmatus*, against HepG2 cells has been described by Tienthanh Nguyen et al. [6] as being related to cell membrane breakdown and mitochondrial malfunction, suppressing cell viability by inducing cell death, cycle arrest, and autophagy. In addition, Ruiyin Guo and colleagues [7] show that the anticancer activity of Smp24, an antimicrobial peptide derived from Egyptian scorpion *Scorpio maurus palmatus*, on A549 cells is associated with the development of apoptosis, autophagy, and cell cycle arrest via dysfunctional mitochondria and ROS buildup. Moreover, Ruiyin Guo et al. [8] have also reported that the production of membrane defects and cytoskeleton disruption by Smp24, a peptide from Egyptian scorpion *Scorpio maurus palmatus*, leads to a strong anticancer effect in a xenograft mouse model of A549. These reports provided new insights into the anticancer mechanism of Smp24, which may aid in the future development of therapy for lung cancer cells.

The transcriptomes of the venom glands of *Mesobuthus martensii* from various populations and genders have been analyzed by Zhiyong Di and colleagues [9]. They have also examined the expression preferences of various toxin gene families as well as the features of members of toxin gene clusters. Their study will encourage further in-depth research and utilization of scorpions and their toxic resources, which will be helpful for establishing standardization in Quanxie identification and medicinal applications in traditional Chinese medicine.

For the first time, Xuekui Nie and colleagues [10] have integrated proteomic and transcriptome methods to examine the variations in venom composition between captive and wild individuals of Chinese cobras. Under breeding conditions, the venom composition differs between captive and wild individuals, demonstrating the plasticity of venom composition. As a result of this study, we will better understand the mechanism of snakebite intoxication and ensure the safe preparation and administration of traditional antivenom and next-generation drugs against snakebites.

As a biocontrol agent, *Pyemotes zhonghuajia* plays a vital role against pests of the Isoptera, Homoptera, Hymenoptera, Lepidoptera, and Coleoptera families. *P. zhonghuajia* injects toxins into the host (eggs, larvae, pupae, and adults), thereby obtaining nourishment for reproduction. By providing a genome assembly of *P. zhonghuajia*, Yanfei Song et al. [11] have given insights into a detailed description of its toxin-related gene families. Their work will contribute to improving the parasitism efficiency of these mites and provide a basis for the development of new biological pesticides.
